# A region within the third extracellular loop of rat Aquaporin 6 precludes trafficking to plasma membrane in a heterologous cell line

**DOI:** 10.1038/s41598-021-93117-8

**Published:** 2021-07-01

**Authors:** D. C. Soler, T. Kowatz, A. E. Sloan, T. S. McCormick, K. D. Cooper, R. Stepanyan, A. Engel, A. Vahedi-Faridi

**Affiliations:** 1 The Department of Neurosurgery, Case Western Reserve University, Cleveland, USA; 2 Brain Tumor and Neuro-Oncology Center, University Hospitals Cleveland Medical Center, Cleveland, USA; 3 Department of Dermatology, Case Western Reserve University, Cleveland, USA; 4 Murdough Family Center for Psoriasis, Case Western Reserve University, Cleveland, USA; 5 Department of Otolaryngology-HNS, Case Western Reserve University, Cleveland, USA; 6 Department of Neurosciences, Case Western Reserve University, Cleveland, USA; 7 Biozentrum, University of Basel, Basel, Switzerland; 8 Department of Physiology and Biophysics, Case Western Reserve University, 10900 Euclid Avenue, Cleveland, OH 44106-4965 USA

**Keywords:** Molecular biology, Structural biology

## Abstract

The inability to over-express Aquaporin 6 (AQP6) in the plasma membrane of heterologous cells has hampered efforts to further characterize the function of this aquaglyceroporin membrane protein at atomic detail using crystallographic approaches. Using an Aquaporin 3-tGFP Reporter (AGR) system we have identified a region within loop C of AQP6 that is responsible for severely hampering plasma membrane expression. Serine substitution corroborated that amino acids present within AQP6^194–213^ of AQP6 loop C contribute to intracellular endoplasmic reticulum (ER) retention. This intracellular retention signal may preclude proper plasma membrane trafficking and severely curtail expression of AQP6 in heterologous expression systems.

## Introduction

Aquaporins are proteins composed of six transmembrane α-helices that form a single water-permeable channel. All monomeric pores exhibit a conserved Asn-Pro-Ala (NPA)^1^ constriction site formed by two short half helical segments and a selectivity filter (SF) at the narrowest point in the channel. All aquaporins are further composed of four monomers, held together by hydrophobic interactions which create a tetramer that displays a central pore^2^. Aquaporins are traditionally divided into two categories: strict water channels and aquaglyceroporins, which in addition to water also transport glycerol and other small solutes via the monomeric pore^3–5^. This classification has expanded considerably over the past decade as more evidence has emerged of potential gas permeability in aquaporins. Boron et al. first demonstrated that Aquaporin 1 (AQP1) expressed in oocytes is also permeable to CO_2_^6^. Molecular dynamic simulations^7^ and monomeric pore inhibitor studies^8^ suggest that approximately half the channel-dependent flux of CO_2_ moves through the four independent water pores, whereas the other half might permeate via the central pore pathway.

Aquaporin 6 is localized mainly in the kidney and it co-localizes with the vH + -ATPase membrane protein. Interestingly, although AQP6 is technically classified as an aquaglycerolporin, its highest sequence homology is more closely related to pure water channels such as AQP0, AQP2 and AQP5. Aquaporin 6 has an additional unique property of being permeable to anions under low (acidic) pH levels^9^. It is therefore surmised that AQP6 may be involved in acid–base regulation^10^. For example, a single amino acid substitution of asparagine by glycine at position 60 (N60G) eliminates AQP6 anion permeability completely when expressed in Xenopus oocytes^11^. Interestingly, this occurs with a concomitant increase of water permeability, similar to AQP2, which is not inhibited by HgCl_2_. Oocytes studies performed by Boron et al.^8,12^ have shown that AQP6 is additionally permeable to NH_3_ as well. Similar N60G mutations in other aquaporins, such as AQP0, AQP1 and AQP2 result instead in a failure of the protein to traffic to the plasma membrane. This strongly suggests that the interaction of transmembrane domains 2 and 5 in AQP6 may result in significant conformational changes^11^ and perturbation of the native protein structure.

Currently, the inability to over-express AQP6 in heterologous systems is a major bottleneck precluding crystallographic studies. AQP6 basal expression in its native location of the kidney is already very low^13^, which makes purification from this source tissue not practicable. Transient transfection of AQP6 in insect or mammalian cell lines has not been successful either^14^.

Like most eukaryotic membrane proteins AQP6 is glycosylated. One potential glycosylation site, N134, is in the region of loop B and may be essential for translocation and function^15^. Therefore, heterologous expression of AQP6 would ideally be carried out in a system which has the ability for post-translational modifications.

In the present work we took advantage of a previously created Aquaporin 3-tGFP (AQP3-tGFP) construct^16^ that displays intense Plasma Membrane (PM) with low cytoplasmic fluorescence, termed the AQP3-tGFP-based Reporter (AGR) system. In the past, we have used this system to identify a novel endoplasmic reticulum (ER)-retention sequence located in the N-terminus of TMC1^17^. We designated these ER-retention sequences “Omega-type” since their presence in a protein leads to a complete termination of PM localization^17^.

Using the AGR system we now have identified a region within the third extracellular loop of AQP6 that decreased PM localization when expressed in HEK293 cells. Using serine-mutagenesis we restored the ability of this region to reach the PM using the AGR system. We hypothesize that the third extracellular loop of AQP6 with the sequence FTGCSMNPARSFGPAVIVGKFAVHWIF harbors an uncharacterized ER retention sequence that may explain the inability to over-express AQP6 in heterologous cell systems.

## Results

### Expression and localization of tagged AQP6 in HEK293 cells

**Figure 1:**
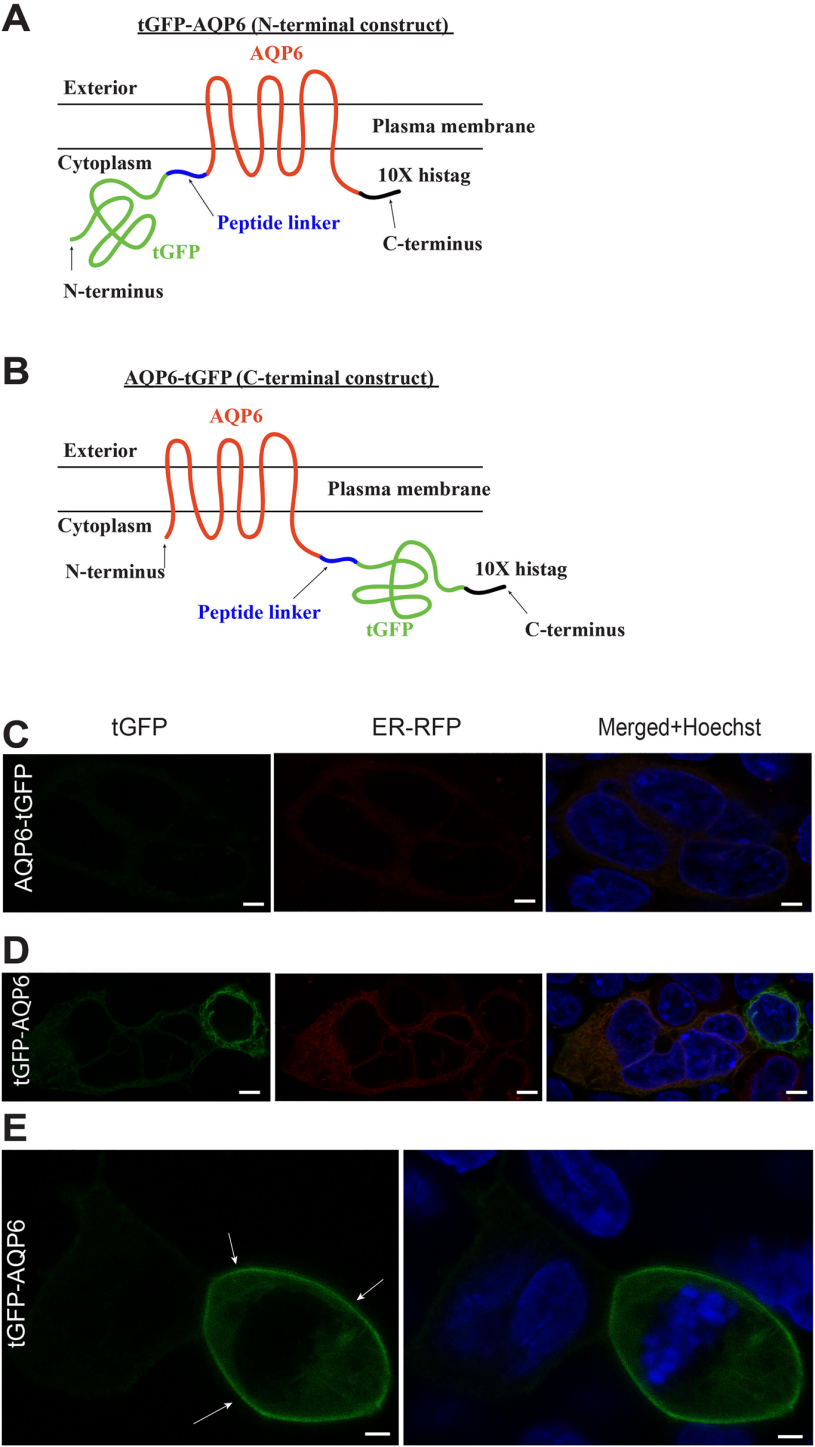
AQP6 expressed in transient heterologous transfection constructs rarely reaches the plasma membrane. (**A**) The N-terminal tGFP-labeled AQP6 construct is shown (tGFP-AQP6). The peptide linker is shown in blue and the C-terminal 10× Histag in black. (**B**) The C-terminal GFP-labeled AQP6 construct is shown (AQP6-tGFP). The peptide linker is shown in blue and the C-terminal 10× Histag in black. (**C**) Transient transfection of an AQP6-tGFP construct in HEK293 cells yields very poor to no expression. (**D**) Transient transfection of tGFP-AQP6 construct in HEK293 cells improves expression but most of the expressed protein is retained in the ER. Scale bars (**C**, **D**): 5 µm. (**E**) Very rarely, the tGFP-AQP6 construct localizes to the PM (white arrows) in HEK293 cells. Scale bar: 3 µm. Cells were observed microscopically using an upright Olympus BX51WI equipped with 100 × 1 NA objective, and images were captured using a Grasshopper3 CMOS camera (FLIR, Richmond, BC, Canada) controlled by Leica LAS X version 3.5 software (available at: https://www.leica-microsystems.com/products/microscope-software/p/leica-las-x-ls/). All figures were created using Adobe Illustrator CS6 (available at: https://adobe.com/products/illustrator), under an Adobe Inc., Creative Cloud Desktop 2019 shared device license to Case Western Reserve University (CWRU) that operates until 3/31/2022.

To study the cellular localization of AQP6 in transiently transfected HEK293 cells, two tagged constructs were made using turbo GFP (tGFP) at the N- or C-terminus of AQP6 (Fig. 1A,B respectively). The C-terminal tGFP tagged construct produced almost no AQP6 expression as previously reported by Ikeda et al.^18^ with a complete absence of plasma membrane (PM) localization (Fig. 1C). Although the N-terminal tGFP tagged version displayed higher levels of expression, localized mainly in the ER (Fig. 1D). However, we were able to identify a small number of transfected cells displaying PM localization (Fig. 1E) confirming previous reports using a different heterologous system^19^. However, in both N- and C-terminal versions, expression levels of AQP6 were so low overall that any attempt to use these constructs for mass-production of AQP6 for structural studies is unlikely.

### Scanning AQP6 residues 1–138 using the AGR system

**Figure 2:**
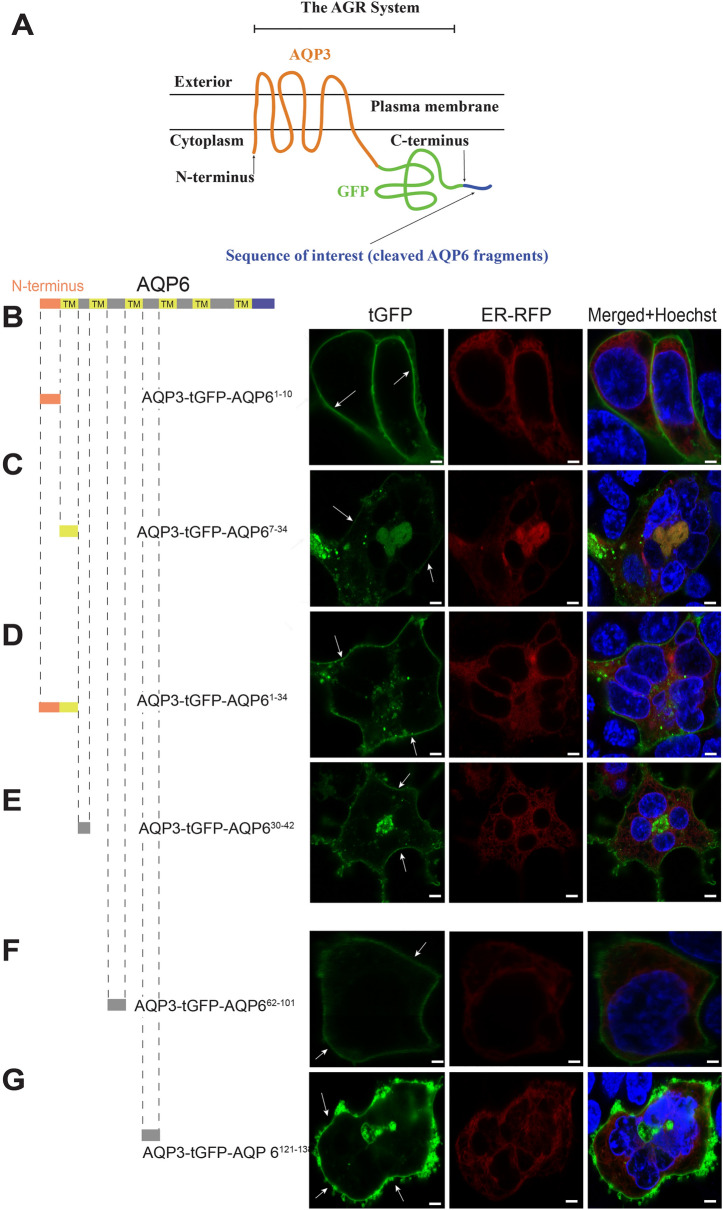
AGR scanning of the first half of AQP6. (**A**) Schematic representation for usage of the AGR system with AQP6 (Figure was adapted from Ref.^17^) under the terms of the Creative Commons CC BY license. (**B**) Attaching the N-terminus of AQP6 (comprising residues AQP6^1–10^) C-terminally to AGR, results in a construct that reaches the PM (white arrows). (**C**) In a similar manner, when the first transmembrane domain (TMD) of AQP6 (comprising residues AQP6^7–34^) is C-terminally attached to AGR, this construct also reaches the PM. (**D**) Using the entire AQP6^1–34^ residues (which include N-terminus and first TMD), instead of the subunits indicated in (**A**, **B**), also results in PM expression when attached to AGR. (**E**) When residues AQP6^30–42^ are used, this construct also localized to the PM. (**F**) Similarly, when peptide residues AQP6^62–101^ are attached to AGR, the construct localized to the PM. Finally, (**G**) when AQP6^121–138^ peptide residues are used the construct also reached the PM. Cells were observed microscopically using an upright Olympus BX51WI equipped with 100 × 1 NA objective and images were captured using a Grasshopper3 CMOS camera (FLIR, Richmond, BC, Canada) controlled by Leica LAS X version 3.5 software (available at: https://www.leica-microsystems.com/products/microscope-software/p/leica-las-x-ls/). All figures were created using Adobe Illustrator CS6 (Available at: https://adobe.com/products/illustrator), under an Adobe Inc., Creative Cloud Desktop 2019 shared device license to Case Western Reserve University (CWRU) that operates until 3/31/2022. Scale bars: 8 µm.

In order to identify potential regions of AQP6 that may be responsible for reducing expression levels (thus containing uncharacterized ER retention or degron motifs^20^) we used our previously described AGR system^17^ (Fig. 2A). Because the AGR system displays very strong PM localization with minimal cytoplasmic staining, any given protein can be cleaved into peptide fragments, and each peptide fragment independently C-terminally attached to AGR to check for its cellular localization potential. If a particular amino acid peptide domain attached to AGR leads to decreased PM localization, it is inferred that this domain may contain an ER-retention motif. If instead PM localization occurs, we then conclude the peptide fragment does not express an Omega-type ER-retention motif. Using the AGR system we tested the intracellular and extracellular loops of AQP6, obviating its transmembrane domains (TMD) except for the initial domain. To our surprise, as shown in Fig. 2, neither AQP6 N-terminal—AQP6^1–10^ domain (Fig. 2B), nor domains AQP6^7–34^ (Fig. 2C), AQP6^1–34^ (Fig. 2D), AQP6^30–42^ (Fig. 2E), AQP6^62–101^ (Fig. 2F), or AQP6^121–138^ (Fig. 2G) precluded PM localization when individually C-terminally attached to the AGR system. However, it is possible our system masked N-terminal targeting signals previously reported for AQP6^18,19^.

### The third outside loop of AQP6 precludes PM localization of AGR

**Figure 3:**
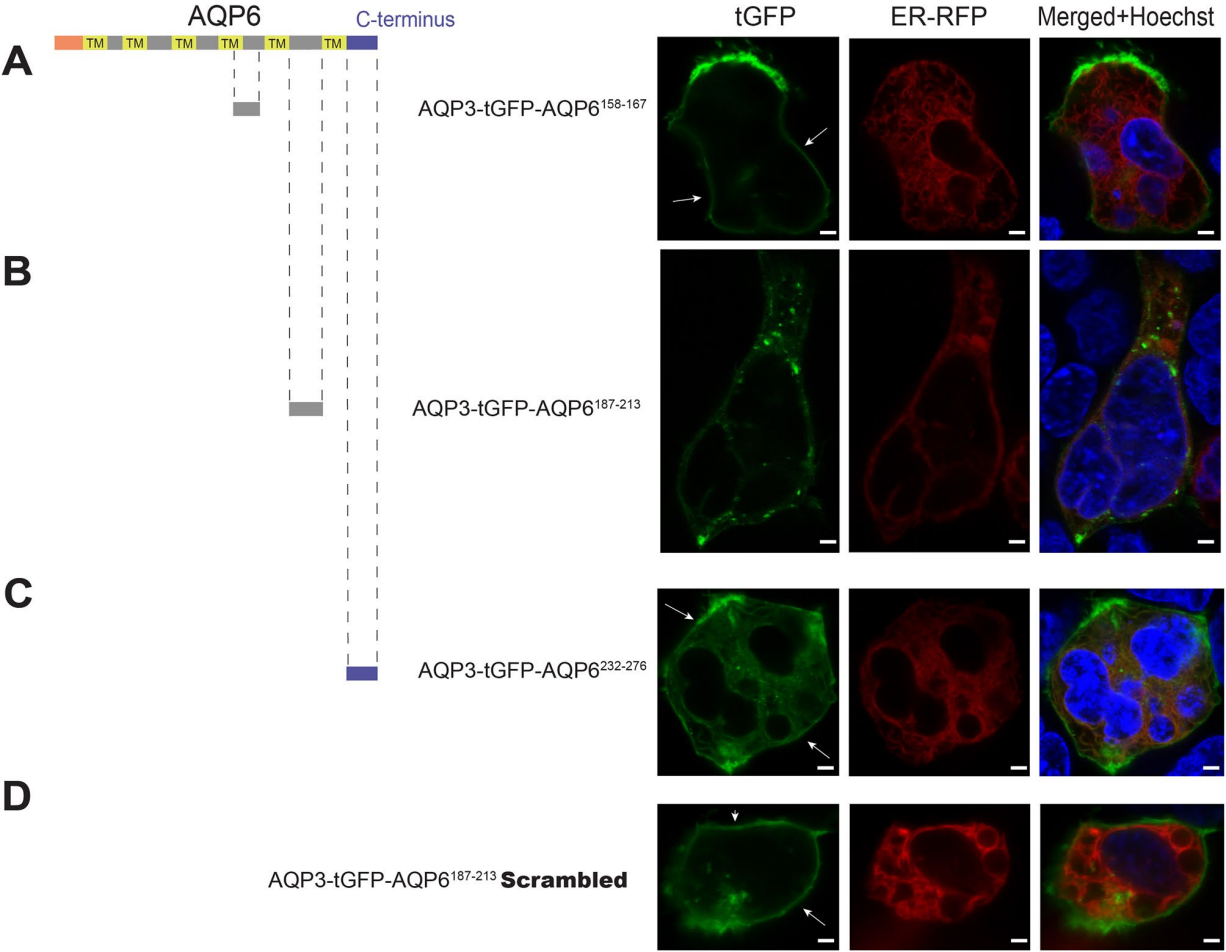
AGR scanning of the second half of AQP6. (**A**) Approaching the second half of AQP6 in a similar manner to Fig. 2, peptide residues AQP6^158–167^ when C-terminally attached to AGR results in a construct that reaches the PM (white arrows). (**B**) However, when residues AQP6^187–213^ are C-terminally attached to AGR, the construct *does not reach the PM*. (**C**) Although, AQP6^232–276^ residues (compromising AQP6 C-terminal) attached to AGR does localize to the PM. (**D**) Interestingly, when a scrambled version of peptide residues AQP6^187–213^ are substituted for the native AQP^187–213^ and C-terminally attached to AGR, this construct is able to reach the PM. Cells were observed microscopically using an upright Olympus BX51WI equipped with 100 × 1 NA objective and images were captured using a Grasshopper3 CMOS camera (FLIR, Richmond, BC, Canada) controlled by Leica LAS X version 3.5 software (available at: https://www.leica-microsystems.com/products/microscope-software/p/leica-las-x-ls/). All figures were created using Adobe Illustrator CS6 (Available at: https://adobe.com/products/illustrator), under an Adobe Inc., Creative Cloud Desktop 2019 shared device license to Case Western Reserve University (CWRU) that operates until 3/31/2022. Scale bars: 8 µm.

We then scanned the rest of the AQP6 loops, individually attaching peptide fragment domains from AQP6^158–167^ (Fig. 3A), AQP6^187–213^ (Fig. 3B) and the C-terminus AQP6^232–276^ domain (Fig. 3C) to the C-terminal of the AGR system. Interestingly, the peptide domain AQP6^187–213^ produced a marked decrease in expression and a total absence of PM localization of the AGR reporter (Fig. 3B). In contrast, a scrambled version of AQP6^187–213^ was able to reach the PM when C-terminally attached to AGR (Fig. 3D), suggesting the presence of an uncharacterized sequence-specific ER retention motif within region AQP6^187–213^.

### Mutagenesis of loop C identifies the retention region

**Figure 4:**
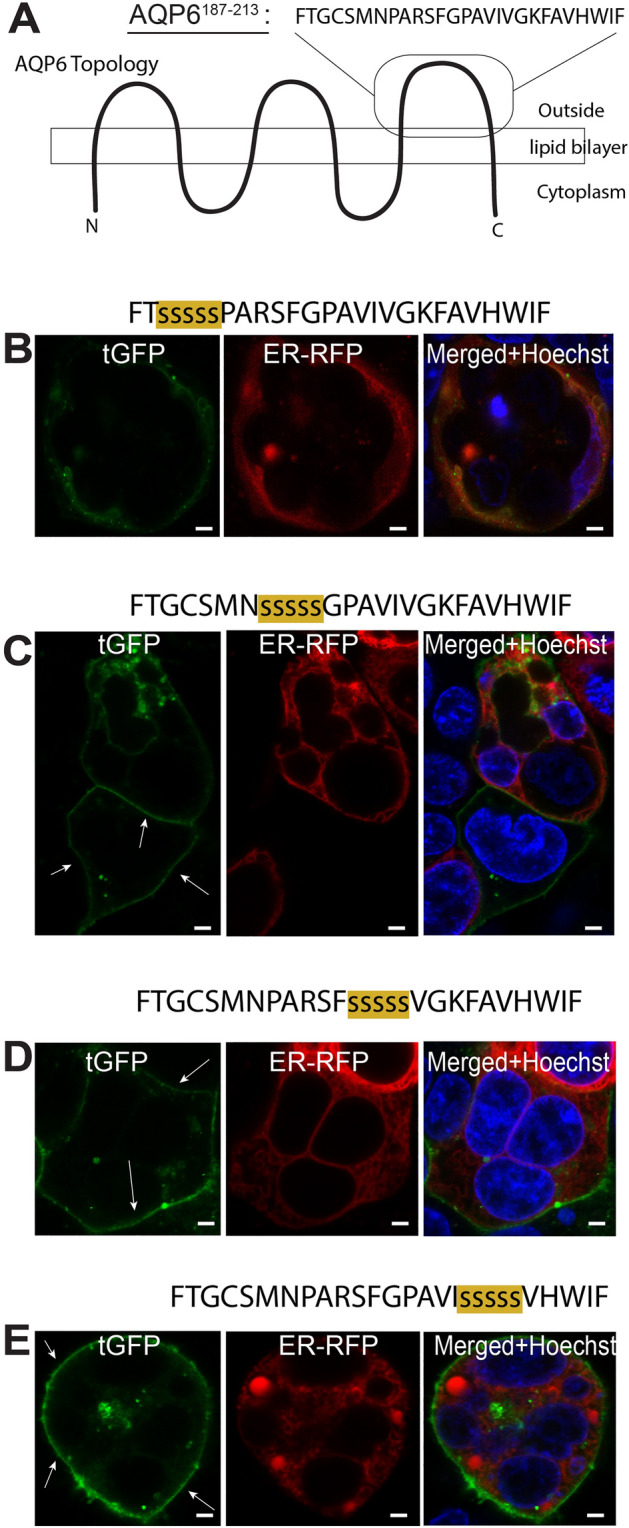
Serine mutagenesis pinpoints Omega signal localization. (**A**) The amino acid sequence comprising peptide fragment AQP6^187–213^ is shown. (**B**) When this peptide fragment was substituted with 5 serine substitutions and C-terminally attached to AGR, this construct fails to localize to the PM of HEK293 cells. In contrast, (**C**–**E**) amino acid serine substitutions within the indicated peptide fragments and then C-terminally attached to AGR, these constructs localize in the PM of HEK293 cells (white arrows). Cells were observed microscopically using an upright Olympus BX51WI equipped with 100 × 1 NA objective and images were captured using a Grasshopper3 CMOS camera (FLIR, Richmond, BC, Canada) controlled by Leica LAS X version 3.5 software (available at: https://www.leica-microsystems.com/products/microscope-software/p/leica-las-x-ls/). All figures were created using Adobe Illustrator CS6 (Available at: https://adobe.com/products/illustrator), under an Adobe Inc., Creative Cloud Desktop 2019 shared device license to Case Western Reserve University (CWRU) that operates until 3/31/2022. Scale bars: 8 µm.

In order to further pinpoint the region that produces the PM targeting in the AGR system, as shown in Fig. 3B, we undertook a systemic serine-mutagenesis approach in order to further elucidate the characteristics of “loop C” (Fig. 4A). Using four different constructs, each C-terminally attached to AGR, we identified the amino acid sequence PARSFGPAVIVGKFAVHWIF as the one responsible for ER retention (Fig. 4B–E).

## Discussion

Polytopic multi-pass membrane proteins are a notoriously difficult to mass express for functional and structural studies^21^. The aquaglyceroporin subtype AQP6 has been known for quite some time to be very difficult to express in heterologous systems^18^. In the present work, we sought to examine the molecular structure of AQP6, focusing on the cellular expression and localization characteristics of intracellular and extracellular AQP6 loops. Using the AGR system^17^, we were able to identify a region contained within the third extracellular loop of AQP6, so called “loop C”, that completely abrogated PM localization, while decreasing the expression levels of the AGR reporter system in HEK293 cells (Fig. 3B). To pinpoint whether or not this ER retention region could be abolished, we used serine-mutagenesis to localize the amino acid sequence containing the ER retention signal. As shown in Fig. 4, five consecutive amino acid substitutions along the underlined sequence FTGCSMNPARSFGPAVIVGKFAVHWIF restored PM localization in the AGR system (Fig. 4C,D), suggesting the retention motif is encoded within the underlined residues. Future studies will address the functionality of this sequence in the native AQP6 protein.

This novel insight may help in further designing a mutagenized AQP6 that displays high-expression levels when transiently expressed in heterologous cells such as HEK293. The ability to overcome the AQP6 ER-retention would improve the chances of producing sufficient quantities of AQP6 to allow crystallographic structural analysis. The AGR system has provided a potential explanation for why AQP6 expression levels remain very low even when heterologously expressed.

The current study does not address why such strong Omega-type ER-retention signals may be present in AQP6. One possible explanation is that proteins expressed in very specialized organs such as the kidneys for AQP6 or mechanosensing organelles such as the stereocilia for TMC1, depend on expression of tissue-specific chaperones that block Omega-type retention signals in order to enforce their tissue-specific effects. Thus attempts to express AQP6 or TMC1 proteins in non-native cell types leave their Omega-type signals exposed, and in the absence of tissue-specific chaperones expressed proteins default to ER-retention. Extended studies of Omega-type signals will attempt to address this hypothesis. Further work will also have to address the question of whether Omega-type ER retention signals can be abrogated using alternative amino acid substitution instead of alanines or serines in order to minimally disrupt the AQP6 native folding configuration.

## Materials and methods

### Plasmid constructs

The AQP3-tGFP-pcDNA5/FRT plasmid was synthesized as previously described^17^. The full cDNA containing human AQP6-tGFP (NP_001643) was synthesized by Biomatik (Wilmington, DE) and tGFP-AQP6 (rat NP_071517.1) by Genscript (Piscatawas, NJ). In the tGFP-AQP6 construct, the tGFP was separated from AQP6 by the following peptide linker which included a Precision Protease site (underlined in blue): AASAVNGSLEVLFQGPAA, and containing *Afe1* and *Hpa1* restriction sites, as well as a 10× C-terminal Histamine Tag (Histag) as shown in Fig. 1A (in black). For the AQP6-tGFP construct, the tGFP was separated from AQP6 by the following peptide linker (Fig. 1B in blue) which included a Precision Protease site: GGSLEVLFQGPAA and a c-terminal 10× Histag (in black) as shown in Fig. 1B.

The AQP6-tGFP construct was subcloned into a pcDNA5/FRT plasmid (Invitrogen, Carlsbad, CA) using restriction enzymes BamH1/EcoRV while the AQP6 was subcloned using Hpa1/EcoRI into a pcDNA5/FRT plasmid containing tGFP. Each construct was custom synthesized by Genscript, codon optimized for mammalian expression in HEK293 cells. Each AQP6-based sequence was then subcloned into the AQP3-tGFP-pcDNA5/FRT plasmid using Hpa1/EcoRV restriction sites. All constructs were confirmed by sequencing.

### Cell culture, transfection

HEK293 cells were purchased from Life Sciences (Carlsbad CA) and seeded in 12-well glass-bottom culture plates (Cellvis, Mountain View, CA) and cultured in DMEM/F12 media (Life Technologies, Carlsbad, CA) supplemented with 10% Fetal Bovine Serum and 1% pen/strep for 3 days prior to transfection. Transfections were performed as described previously^17^. Briefly, on the day of transfection, spent media was replaced by fresh media and 500 ng of plasmid constructs were transfected into HEK293 cells using Lipofectamine 2000 (Life Technologies, Carlsbad, CA) following the manufacturer’s instructions. Red Fluorescent Protein based BacMam 2.0 constructs specific for ER were co-transfected the same day following the manufacturer’s instructions (Thermo Fisher Scientific, Waltham, MA). The cell nuclei was labelled using Hoechst dye (Life Technologies, CA). All recorded experiments were performed 48 h after transfection.

### Imaging

Transfected HEK293 cells were examined with an SP8 confocal fluorescence microscope using 63 × 1.4 NA objective, Leica (Wetzlar, Germany) as described previously^17^. Briefly, cells were observed microscopically using an upright Olympus BX51WI equipped with 100 × 1 NA objective and images were captured using a Grasshopper3 CMOS camera (FLIR, Richmond, BC, Canada) controlled by Leica LAS X version 3.5 software (available at: https://www.leica-microsystems.com/products/microscope-software/p/leica-las-x-ls/). All figures were created using Adobe Illustrator CS6 (Available at: https://adobe.com/products/illustrator), under an Adobe Inc., Creative Cloud Desktop 2019 shared device license to Case Western Reserve University (CWRU) that operates until 3/31/2022.

## References

[1] Vahedi-Faridi A, Engel A (2016). Aquaporin structure and selectivity. Aquaporins in Health and Disease: New Molecular Targets for Drug Discovery.

[2] Gonen T, Walz T (2006). The structure of aquaporins. Q. Rev. Biophys..

[3] Verkman AS (2008). Mammalian aquaporins: Diverse physiological roles and potential clinical significance. Expert Rev. Mol. Med..

[4] Wu B, Beitz E (2007). Aquaporins with selectivity for unconventional permeants. Cell Mol. Life Sci..

[5] Heymann JB, Engel A (1999). Aquaporins: Phylogeny, structure, and physiology of water channels. News Physiol. Sci..

[6] Nakhoul NL, Davis BA, Romero MF, Boron WF (1998). Effect of expressing the water channel aquaporin-1 on the CO_2_ permeability of Xenopus oocytes. Am. J. Physiol..

[7] Wang Y, Cohen J, Boron WF, Schulten K, Tajkhorshid E (2007). Exploring gas permeability of cellular membranes and membrane channels with molecular dynamics. J. Struct. Biol..

[8] Musa-Aziz R, Chen LM, Pelletier MF, Boron WF (2009). Relative CO_2_/NH_3_ selectivities of AQP1, AQP4, AQP5, AmtB, and RhAG. Proc. Natl. Acad. Sci. USA..

[9] Yasui M, Hazama A, Kwon TH, Nielsen S, Guggino WB, Agre P (1999). Rapid gating and anion permeability of an intracellular aquaporin. Nature.

[10] Yasui M (2009). pH regulated anion permeability of aquaporin-6. Handb. Exp. Pharmacol..

[11] Verkman AS (2012). Aquaporins in clinical medicine. Annu. Rev. Med..

[12] Geyer RR, Musa-Aziz R, Qin X, Boron WF (2013). Relative CO(2)/NH(3) selectivities of mammalian aquaporins 0–9. Am. J. Physiol. Cell Physiol..

[13] Yasui M, Kwon TH, Knepper MA, Nielsen S, Agre P (1999). Aquaporin-6: An intracellular vesicle water channel protein in renal epithelia. Proc. Natl. Acad. Sci. USA..

[14] Eifler N, Duckely M, Sumanovski LT (2007). Functional expression of mammalian receptors and membrane channels in different cells. J. Struct. Biol..

[15] Krueger, A. Expression and purification of aquaporin-6 in different systems. *Thesis dissertation* (2012).

[16] Soler DC, Young AE, Vahedi-Faridi A, McCormick TS (2018). Generation of Flp-in(tm)-ready DG44 and Lec 3.2.8.1 CHO cell lines for quick and easy constitutive protein expression. Biotechniques.

[17] Soler DC, Manikandan M, Gopal SR, Sloan AE, McCormick TS, Stepanyan R (2019). An uncharacterized region within the N-terminus of mouse TMC1 precludes trafficking to plasma membrane in a heterologous cell line. Sci. Rep..

[18] Ikeda M, Beitz E, Kozono D, Guggino WB, Agre P, Yasui M (2002). Characterization of aquaporin-6 as a nitrate channel in mammalian cells. Requirement of pore-lining residue threonine 63. J. Biol. Chem..

[19] Beitz E, Liu K, Ikeda M, Guggino WB, Agre P, Yasui M (2006). Determinants of AQP6 trafficking to intracellular sites versus the plasma membrane in transfected mammalian cells. Biol Cell..

[20] Cho S, Dreyfuss G (2010). A degron created by SMN2 exon 7 skipping is a principal contributor to spinal muscular atrophy severity. Genes Dev..

[21] Carpenter EP, Beis K, Cameron AD, Iwata S (2008). Overcoming the challenges of membrane protein crystallography. Curr. Opin. Struct. Biol..

